# Impact of Tacrolimus Trough Levels at Discharge on Early Post-Kidney Transplantation Outcomes: A Nationwide Cohort Study

**DOI:** 10.3390/jcm14165707

**Published:** 2025-08-12

**Authors:** Heungman Jun, Young Ju Oh, Hyo Kee Kim, Jun Young Lee, Yeong Hoon Kim, Joong Kyung Kim, Jaeseok Yang, Myoung Soo Kim, Cheol Woong Jung, Korean Organ Transplantation Registry Study Group

**Affiliations:** 1 Division of Transplantation and Vascular Surgery, Department of Surgery, Korea University Anam Hospital, Korea University College of Medicine, 73 Koryodae-ro, Seongbuk-gu, Seoul 02841, Republic of Korea; midasia@hanmail.net (H.J.); yjblue816@naver.com (Y.J.O.); 2 Department of Surgery, Korea University Guro Hospital, Korea University College of Medicine, Seoul 08308, Republic of Korea; gogohyohyo@gmail.com; 3 Department of Nephrology, Yonsei University Wonju College of Medicine, Wonju Severance Christian Hospital, Wonju 26426, Republic of Korea; junyoung07@yonsei.ac.kr; 4 Department of Internal Medicine, Inje University Busan Paik Hospital, Busan 47392, Republic of Korea; yeonghnl@inje.ac.kr; 5 Department of Internal Medicine, Bongseng Memorial Hospital, Busan 48773, Republic of Korea; 6 Division of Nephrology, Department of Internal Medicine, Severance Hospital, Yonsei University College of Medicine, Seoul 03722, Republic of Korea; jcyjs@yuhs.ac; 7 Department of Surgery, Severance Hospital, Yonsei University College of Medicine, Seoul 03722, Republic of Korea; ysms91@yuhs.ac

**Keywords:** tacrolimus, kidney transplantation, immunosuppressive agents, graft rejection

## Abstract

**Introduction**: Tacrolimus is a cornerstone immunosuppressant in kidney transplantation (KT), but its narrow therapeutic index necessitates precise monitoring. Early post-transplant tacrolimus trough concentrations (C0) are critical, as suboptimal levels can increase rejection and infection risks. This study evaluated the impact of C0 levels at discharge on early post-transplant outcomes in a large Korean cohort. **Materials and Methods**: This retrospective analysis included 5293 KT recipients from the Korean Organ Transplant Registry (KOTRY) who received a kidney transplant between 2014 and 2019. Recipients were categorized into three groups based on C0 levels at discharge: <5.9 ng/mL, 5.9–9.5 ng/mL, and >9.5 ng/mL. Clinical outcomes, including serum creatinine (sCr), biopsy-proven acute rejection (BPAR), and infections requiring hospitalization, were analyzed using the Kruskal–Wallis test and chi-squared test. **Results**: The BPAR rates were 22.5%, 20.9%, and 21.5% for the low, middle, and high C0 groups, respectively (*p* = 0.221). However, the incidence of infections requiring hospitalization was significantly higher in the high C0 group (28.1%) compared to the middle (23.9%) and low (21.7%) groups at 1-year follow-up (*p* < 0.001). In high-risk recipients, lower C0 levels correlated with increased BPAR rates (33.9% vs. 29.1% and 26.4%, *p* = 0.030). Higher intrapatient variability (IPV) between discharge and 6 months was linked to higher infection risk in all recipients and increased BPAR and infection risk in high-risk patients. **Conclusions**: Optimal C0 levels at discharge are essential to balance rejection and infection risks in KT. Lower C0 levels and higher IPV increase the risk of adverse outcomes, especially in high-risk sensitized recipients, underscoring the need for careful monitoring and personalized management.

## 1. Introduction

Tacrolimus is the cornerstone of modern immunosuppressive regimens, and is widely used to maintain stable immunosuppression in kidney transplantation (KT). However, its potential toxicity has led to increasing efforts to minimize its use [1]. Currently, the most common regimen combines tacrolimus with mycophenolate derivatives and steroids [2]. Maintaining an appropriate dosage is critical for balancing efficacy and minimizing side effects, making therapeutic drug monitoring essential. Tacrolimus has a narrow therapeutic index, nonlinear pharmacokinetics, and high interindividual variability influenced by genetic polymorphisms (e.g., CYP3A4, CYP3A5), drug–food interactions, and co-medications, making direct blood concentration monitoring essential [3]. Tacrolimus trough concentrations (C0), measured immediately before the next dose, are commonly used for therapeutic drug monitoring because they correlate well with the area under the concentration curve (AUC), reflecting overall drug exposure. As a practical surrogate for AUC, C0 levels help guide dose adjustments to maintain immunosuppressive efficacy while minimizing the risk of toxicity and graft rejection, ensuring optimal dosing in clinical practice [4].

An appropriate tacrolimus concentration during the first year after KT is particularly critical for preventing complications. Lower tacrolimus levels in the early post-transplant period have been associated with the development of de novo donor-specific anti-HLA antibodies (DSAs), which increase the risk of rejection [5]. Gaynor emphasized that C0 levels below 4.0 ng/mL should be avoided during the first year to prevent acute rejection [6]. The KDIGO guidelines recommend maintaining a C0 of 10 ng/mL for early standard dosing; however, many patients are discharged with lower concentrations, suggesting insufficient immunosuppression. This issue is particularly concerning, as hospital stays shorten, and it is especially important in highly sensitized recipients, in whom optimal tacrolimus levels are crucial for preventing rejection [7]. This study aims to explore the impact of C0 levels at discharge on early post-transplant complications, including rejection and infections, by analyzing data from a large-scale Korean registry.

## 2. Methods

### 2.1. Study Design and Ethical Considerations

This study utilized data from the Korean Organ Transplant Registry (KOTRY), a nationwide cohort established in 2014. KOTRY collects longitudinal demographic and clinical data from donors and recipients of kidney, liver, pancreas, heart, and lung transplants across 59 centers in South Korea [8].

**Figure 1:**
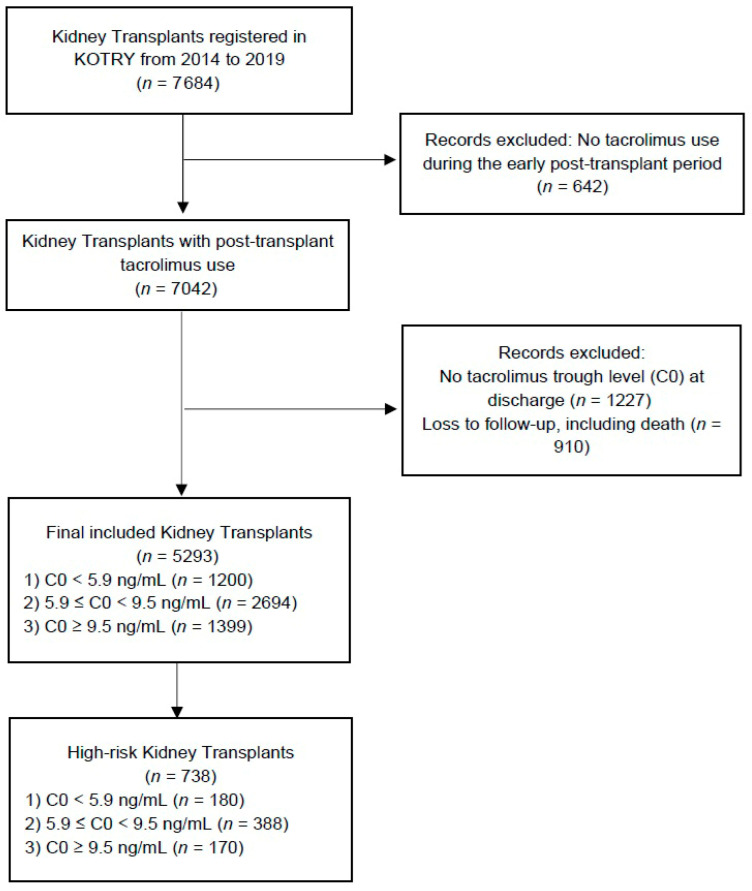
Population flow diagram of the study cohort.

The study population consists of 5293 KT recipients from the KOTRY database, spanning from 30 April 2014 to 31 December 2019. The study was a retrospective analysis of data from the KOTRY database. The inclusion criteria were as follows: recipients who used tacrolimus continuously from the early post-transplant period for at least one year, recipients aged 18 or older, and those with a follow-up duration of at least one year. Records were excluded based on the following criteria: no tacrolimus use during the early post-transplant period, absence of C0 levels at discharge, and loss to follow-up, including death. The final study population was divided into three groups based on C0 levels at discharge, defined by population percentiles: recipients with C0 levels below 5.9 ng/mL (below the 25th percentile), those with C0 levels between 5.9 ng/mL and 9.5 ng/mL (between the 25th and 75th percentiles), and those with C0 levels above 9.5 ng/mL (above the 75th percentile). Recipients who had a positive crossmatch, a panel-reactive antibody (PRA) of 10% or higher, or positive DSA were deemed to be part of a high-risk group. The population flow diagram of this study is presented in Figure 1.

The study was approved by the Institutional Review Board of Korea University Anam Hospital (IRB No. 2014AN0272; approval date: 16 June 2014) and conducted in accordance with the Declarations of Helsinki (1975, revised in 2013) and Istanbul. Data were provided upon approval of the investigator’s proposal and excluded personal information. Informed consent was obtained from both donors and recipients involved in the study.

### 2.2. Data Collection and Clinical Outcomes

Data were collected from the KOTRY database. The collected data included information on recipients’ gender, age, body weight, diabetes status, and high-risk group status. For donors, data on gender, age, diabetes status, deceased donor status, and re-transplantation were gathered. Additional information included delayed graft function (DGF) in recipients. The study focused on recipients who used tacrolimus from the early post-transplant period. The selection of induction agents, tacrolimus dosing, and target C0 levels was determined based on each patient’s clinical status and institutional protocols. Tacrolimus trough levels were measured using a chemiluminescent microparticle immunoassay, a method widely used in Korean hospitals. C0 levels were measured 12 h after the previous dose and monitored at least monthly during the first 6 months, with more frequent assessments and dose adjustments as needed, particularly in cases of persistently low C0 levels. Immunosuppressive therapy data were also obtained; these related to the use of antithymocyte globulin (ATG) as an induction agent, the daily tacrolimus dose at discharge, and the C0.

The outcomes assessed included C0 levels and serum creatinine (sCr) levels at 6 months and 1 year, as well as biopsy-proven acute rejection (BPAR) at 1 year. Both protocol biopsies, performed based on institutional practices, and indication biopsies, conducted when clinical rejection was suspected, were used to identify forms of acute rejection. BPAR was diagnosed when treatment was initiated for confirmed rejection in both cases. Infections were defined as those requiring hospitalization. While conventional calculations of intrapatient variability (IPV) of C0 levels require multiple measurements, we adopted a simplified method using two time points. IPV was calculated as the absolute difference between tacrolimus trough levels at discharge and 6 months, divided by the average of the two values. This approach was adapted from previous methods assessing variability between limited time points [9].

### 2.3. Statistical Analysis

The normality of data distribution was assessed using the Shapiro–Wilk test. Since the data did not meet normality assumptions, the three groups based on C0 levels were compared using the Kruskal–Wallis one-way analysis for continuous variables. Categorical variables were compared using Pearson’s chi-squared test. Cox proportional hazards regression was performed to evaluate the association between C0 variation and the risk of BPAR and infections, with hazard ratios (HRs) and 95% confidence intervals (CIs) calculated for both the overall and high-risk groups. A *p*-value < 0.05 was considered statistically significant. All statistical analyses were conducted using SPSS software, version 25 (IBM Corp, Armonk, NY, USA).

## 3. Results

### 3.1. Patient Characteristics

Among the 5293 enrolled KT recipients, 59.6% were male, with a mean age of 49.05 ± 11.47 years; 30.2% of the enrolled recipients had diabetes. Donors were 52.5% male, with a mean age of 47.27 ± 12.82 years, and 5.0% had diabetes. Deceased donor KTs accounted for 36.0%, and 7.4% were retransplants. DGF occurred in 3.7%, and 13.9% were classified as high-risk. The baseline characteristics according to C0 groups at discharge (<5.9, 5.9–9.5, ≥9.5 ng/mL) are summarized in Table 1. Male recipients and deceased donor KTs were significantly more frequent in the high C0 group (*p* = 0.012 and *p* < 0.001, respectively). High-risk recipients tended to be more common in the low C0 group, but this was not statistically significant (*p* = 0.069).

ATG induction was slightly more frequent in the low C0 group without statistical significance. Mean tacrolimus daily doses and C0 levels at discharge, 6 months, and 1 year differed significantly among the groups (all *p* < 0.001, Table 2).

### 3.2. Outcomes in Overall and High-Risk Groups

sCr levels at 6 months and 1 year showed no significant differences among the C0 groups. The incidence of infections requiring hospitalization was significantly higher in the high C0 group at 1 year (28.1%) compared to the middle (23.9%) and low (21.7%) groups (*p* < 0.001). BPAR rates did not differ significantly between groups in the overall population (*p* = 0.221) (Table 2).

In the high-risk subgroup (Table 3), sCr levels at discharge and 1 year were similar across C0 groups. However, BPAR incidence was significantly higher in the low C0 group (33.9%) compared to the middle and high C0 groups (29.1% and 26.4%, respectively; *p* = 0.030). Infection rates in the high-risk group did not differ significantly among C0 groups (*p* = 0.255).

### 3.3. IPV in C0 Levels Between Discharge and 6 Months

The authors estimated early post-transplant IPV using a simplified method based on tacrolimus trough levels at discharge and 6 months (Table 4). In the overall group, higher IPV was significantly associated with an increased risk of infections (HR 1.049, 95% CI 1.024–1.075, *p* < 0.001), but not with BPAR (*p* = 0.191). In the high-risk group, higher IPV was significantly linked to increased risks of both BPAR (HR 1.110, 95% CI 1.037–1.188, *p* = 0.003) and infections (HR 1.085, 95% CI 1.014–1.162, *p* = 0.019).

## 4. Discussion

### 4.1. Efforts to Achieve Optimal C0 Levels

Efforts to achieve optimal C0 indicate that tacrolimus is essential for preventing acute rejection in KT, but its potential toxicity necessitates careful monitoring to maintain appropriate levels [1]. IPV in tacrolimus pharmacokinetics is driven by genetic polymorphisms in metabolizing enzymes (CYP3A4, CYP3A5) and transporters (ABCB1/P-glycoprotein). These differences affect drug metabolism and clearance, resulting in variable blood levels and clinical outcomes such as graft survival and toxicity [3]. Lower C0 levels in the first 12 months are linked to an increased risk of acute rejection [6]. Research, including the Symphony and ELITE trials, demonstrates that maintaining C0 levels of 5–10 ng/mL while minimizing calcineurin inhibitors improves graft survival and reduces adverse effects [10]. Optimizing tacrolimus use enhances graft and patient outcomes, supporting long-term transplantation success. As shown in Table 2, while C0 levels at discharge exhibited significant variability, they gradually converged to values between 6 and 7 ng/mL by 6 months and 1 year. There was no significant difference in BPAR among the three C0 level groups. However, the group with higher C0 levels experienced an increased incidence of infections requiring hospitalization. The authors noted that while C0 levels stabilized over time, excessive tacrolimus use in the early period actually led to more complications without added benefits.

### 4.2. Optimal C0 Levels in Sensitized High-Risk Groups

Optimal C0 levels in sensitized high-risk groups are particularly important, as DSA and a positive flow cytometry crossmatch increase the risk of antibody-mediated rejection in KT [11]. While historically linked to inferior outcomes, KT in highly sensitized recipients has become increasingly viable with advances in desensitization and post-transplant management. Despite the complexity of these cases, allograft survival rates have been acceptable, with recent cohorts showing promising long-term outcomes [12]. These findings support KT as a feasible option for highly sensitized recipients facing prolonged dialysis and limited donor availability [11].

Particularly in sensitized recipients, careful monitoring of C0 levels is crucial. Richards et al. reported that concentrations of 8 ng/mL are necessary to reduce early acute rejection [7]. Another study found that sensitized patients had twice the incidence of biopsy-proven antibody-mediated rejection and acute cellular rejection compared to nonsensitized patients [13]. In this study, the sensitized high-risk group was analyzed separately, and it was observed that the group with lower C0 levels at discharge had a higher incidence of BPAR within the first year. Interestingly, the incidence of infection requiring hospitalization was not higher in the high-C0 group. The authors suggest this may be related to the frequent use of intravenous immunoglobulin for desensitization and valganciclovir for cytomegalovirus prophylaxis in sensitized recipients. The authors emphasized that the initial C0 level is particularly critical in high-risk groups and suggested that identifying appropriate cutoff values for C0 levels in different regions could be an important area for future research.

### 4.3. The Role of IPV and C0 Levels at Discharge in Post-Transplant Outcomes

Maintaining stable immunosuppressive levels is crucial to prevent rejection and toxicity [14]. High IPV in tacrolimus concentrations at 3 months post-transplant has been linked to increased risks of fibrosis, tubular atrophy, and chronic lesion progression [15]. These findings suggest that IPV may be a modifiable risk factor, highlighting the importance of consistent monitoring.

Hospital stays after transplantation have decreased in recent years. A review by Prionas et al. reported an average stay of 18–21 days in 2010, compared to 5–7 days in 2018–2019, despite center differences [16]. As hospital stays shorten, achieving stable C0 levels before discharge becomes more challenging, particularly since induction therapy is initiated first and maintenance therapy is adjusted within the first few postoperative days. Due to this variability, Alghenem et al. found that fewer than 40% of patients reached target tacrolimus levels during the first month post-transplant [17]. Yin et al. emphasized the importance of maintaining stable, low IPV during this period to prevent acute rejection [18]. Richards et al. demonstrated that achieving a tacrolimus trough concentration of ≥8 ng/mL early after transplant is associated with reduced BPAR in moderately sensitized recipients [7], underscoring the importance of early and discharge-level monitoring.

Building on these findings, the present study evaluated both C0 levels at discharge and subsequent IPV. In the high-risk group, higher IPV between discharge and 6 months was associated with increased risk of BPAR and infections. The authors suggest that both discharge C0 levels and early IPV may have predictive value in transplant outcomes and warrant further investigation.

### 4.4. Limitations

This study had several limitations. The analysis was limited to a one-year period, which may not capture long-term allograft outcomes, and key survival endpoints such as patient and graft survival were not included. Data collection from multiple centers introduced variability in immunosuppressant protocols, C0 monitoring, hospitalization duration, and infectious event assessments due to differing diagnostic criteria and incomplete pathogen identification. The reasons for performing biopsies were not clearly defined, and rejection classifications lacked standardization. Although this study highlights the clinical relevance of C0 levels at discharge, it did not establish a clear link with IPV or provide specific dosing recommendations. In addition, IPV was estimated using only two time points due to the study’s time constraints, which may not fully reflect the true variability. Although the large sample size enhances external validity, the narrow focus on graft rejection as the primary endpoint may introduce bias by excluding other important clinical outcomes, thereby limiting a comprehensive assessment of tacrolimus’s clinical impact. While more recent data are available, the 2014–2019 dataset was used due to its completeness and availability of one-year follow-up data. The large-scale, multicenter nature of the study also required substantial time for data processing, limiting inclusion of newer cases.

## 5. Conclusions

Achieving optimal C0 levels at discharge is crucial for balancing rejection and infection risks in KT. In high-risk sensitized recipients, lower C0 levels were associated with an increased risk of acute rejection, while higher IPV was linked to both acute rejection and infection. These findings highlight the need for meticulous and consistent monitoring, particularly in this vulnerable group. Developing individualized protocols and target thresholds is essential to improving outcomes.

## Figures and Tables

**Table 1:** Baseline characteristics according to tacrolimus trough levels at discharge following kidney transplantation.

Variables	C0 < 5.9 ng/mL (*n* = 1200)	5.9 ≤ C0 < 9.5 ng/mL (*n* = 2694)	C0 ≥ 9.5 ng/mL (*n* = 1399)	*p*-Value
Male (R)	695 (57.9)	1608 (59.9)	883 (63.1)	0.012
Age, years (R)	49.28 ± 11.45	49.46 ± 11.43	49.89 ± 11.83	0.402
Body Weight, kg (R)	63.74 ± 34.42	65.05 ± 36.38	65.51 ± 39.20	0.512
Diabetes (R)	349 (29.1)	819 (30.4)	433 (30.9)	0.569
Male (D)	610 (50.9)	1441 (53.5)	722 (51.6)	0.215
Age, years (D)	46.55 ± 12.70	47.92 ± 12.98	48.31 ± 12.87	0.201
Diabetes (D)	54 (4.6)	135 (5.1)	77 (5.6)	0.469
Deceased donor	369 (30.7)	1021 (37.9)	523 (37.3)	<0.001
Re-transplantation	95 (7.9)	208 (7.7)	99 (7.1)	0.702
Delayed Graft Function	52 (4.3)	99 (3.9)	46 (3.3)	0.361
High-Risk Group *	180 (15.0)	388 (14.4)	170 (12.2)	0.069
sCr, at discharge, mg/dL	1.20 ± 0.71	1.18 ± 0.59	1.17 ± 0.49	0.320

Data are expressed as numbers (%) and means ± SDs. C0—tacrolimus trough concentration; R—recipients’; D—donors’; sCr—serum creatinine level. * The high-risk group is defined as having a positive crossmatch, panel-reactive antibody ≥10%, or positive donor-specific HLA antibodies.

**Table 2:** Immunosuppressive therapy and outcomes based on tacrolimus trough levels at discharge following kidney transplantation.

Variables	C0 < 5.9 ng/mL (*n* = 1200)	5.9 ≤ C0 < 9.5 ng/mL (*n* = 2694)	C0 ≥ 9.5 ng/mL (*n* = 1399)	*p*-Value
ATG induction	287 (23.9)	580 (21.5)	280 (20.0)	0.053
Tacrolimus daily dose at discharge, mg	5.25 ± 2.84	6.31 ± 3.71	7.13 ± 4.13	<0.001
MMF usage at discharge	1110 (92.5)	2518 (93.4)	1305 (93.2)	0.354
C0 at discharge, ng/mL	4.43 ± 1.07	7.59 ± 1.00	11.67 ± 2.69	<0.001
C0 at 6 months, ng/mL	5.98 ± 2.28	6.85 ± 2.39	7.44 ± 2.58	<0.001
C0 at 1 year, ng/mL	6.05 ± 2.49	6.60 ± 2.33	6.72 ± 2.34	<0.001
sCr, at 6 months, mg/dL	1.23 ± 0.51	1.26 ± 1.45	1.32 ± 1.67	0.497
sCr, at 1 year, mg/dL	1.22 ± 0.58	1.22 ± 0.56	1.23 ± 0.48	0.833
BPAR	271 (22.5)	564 (20.9)	302 (21.5)	0.221
Infections *	261 (21.7)	645 (23.9)	393 (28.1)	<0.001

Data are expressed as numbers (%) and means ± SDs. C0—tacrolimus trough concentration; ATG—antithymocyte globulin; MMF—mycophenolate mofetil; sCr—serum creatinine level; BPAR—biopsy-proven acute rejection. * Infections refer to those requiring hospitalization.

**Table 3:** Outcomes in the high-risk group * based on tacrolimus trough levels at discharge following kidney transplantation.

Variables	C0 < 5.9 ng/mL (*n* = 180)	5.9 ≤ C0 < 9.5 ng/mL (*n* = 388)	C0 ≥ 9.5 ng/mL (*n* = 170)	*p*-Value
sCr, at discharge, mg/dL	1.12 ± 0.72	1.06 ± 0.46	1.03 ± 0.43	0.269
sCr, at 1 year, mg/dL	1.14 ± 0.64	1.14 ± 0.51	1.08 ± 0.29	0.483
BPAR	61 (33.9)	113 (29.1)	45 (26.4)	0.030
Infections **	48 (26.7)	106 (27.3)	50 (29.4)	0.255

Data are expressed as numbers (%) and means ± SDs. C0—tacrolimus trough concentration; sCr—serum creatinine level; BPAR—biopsy-proven acute rejection. * The high-risk group is defined as having a positive crossmatch, panel-reactive antibody ≥10%, or positive donor-specific HLA antibodies. ** Infections refer to those requiring hospitalization.

**Table 4:** Predictive impact of intrapatient variability in C0 levels between discharge and 6 months on BPAR and infections in overall and high-risk groups.

Characteristics	Hazard Ratio	95% CI	*p* Value
For Overall Group			
BPAR	1.017	0.991–1.044	0.191
Infections *	1.049	1.024–1.075	<0.001
For High-Risk Group			
BPAR	1.110	1.037–1.188	0.003
Infections *	1.085	1.014–1.162	0.019

C0—tacrolimus trough concentration; BPAR—biopsy-proven acute rejection; CI—confidence interval. * Infections refer to those requiring hospitalization.

## Data Availability

The data that support the findings of this study are available from the Korean Organ Transplantation Registry (KOTRY), but the availability of these data is restricted, as they were used under license for the current study and are not publicly available. However, the data may be made available upon reasonable request and with permission from the KOTRY committee.

## Supplementary Materials

The following supporting information can be downloaded at: https://www.mdpi.com/article/10.3390/jcm14165707/s1. Collaborators/Membership of the Group/Team Name is provided in the Supplementary Materials.
