# Supplementary material for: Impact of Tacrolimus Trough Levels at Discharge on Early Post-Kidney Transplantation Outcomes: A Nationwide Cohort Study
Source: J Clin Med. 2025 Aug 12;14(16):5707. doi: 10.3390/jcm14165707 (PMC12387067; doi:10.3390/jcm14165707)
Supplement: Supplementary file 1 [file jcm-14-05707-s001.zip › jcm-3759938-supplementary.pdf]

KOTRY STUDY GROUP.

Jaeseok Yang[1]: Myoung Soo Kim[2], Ji Yoon Choi[3], Cheol Woong Jung[4], Jun Young Lee[5], Yeong Hoon Kim[6], Joong Kyung Kim[7], Chan-Duck Kim[8], Eun Jeoung Ko[9], Sik Lee[10], Yeon Ho Park[11], Su Hyung Lee[12], Jae Berm Park[13], Jung Hwan Park[14], Seok Hui Kang[15], Tae Hyun Ban[16], Sang Heon Song[17], Seung Hwan Song[18], Ho Sik Shin[19], Byung Ha Chung[20], Hye Eun Yoon[21], Ki-Ryang Na[22], Dong Ryeol Lee[23], Dong Won Lee[24], Jieun Oh[25], Su Woong Jung[26], Yu Ho Lee[27], Hyejin Mo[28], Jeong-Hoon Lee[29], Jin Seok Jeon[30], Sang Youb Han[31], Jin Sug Kim[32], Jong Soo Lee[33], Man Ki Ju[34], Jong Cheol Jeong[35], Soo Jin Na Choi[36], Sung Shin[37], Seungyeup Han[38], Kyu Ha Huh[39], Seun Deuk Hwang[40], Sangil Min[41,] Young Soo Chung[42], Young Joo Kwon[43.]

[1]Division of Nephrology: Department of Internal Medicine, Yonsei University College of Medicine, Severance Hospital.

[2] Department of Surgery: Severance Hospital, Yonsei University College of Medicine.

[3] Department of Surgery: College of Medicine, Han Yang University, Seoul, Korea.

[4] Department of Surgery: Korea University Anam Hospital.

[5] Department of Nephrology, Yonsei University Wonju College of Medicine, Wonju Severance Christian Hospital.

[6] Department of Internal Medicine, Inje University Busan Paik Hospital.

[7] Department of Internal Medicine, Bongseng Memorial Hospital.

[8] Department of Internal Medicine, School of Medicine, Kyungpook National University Hospital.

[9] Division of Nephrology: Department of Internal Medicine, Bucheon St. Mary's Hospital.

[10] Department of Internal Medicine, Jeonbuk National University Hospital.

[11] Department of Surgery: Gil Medical Center, Gachon University College of Medicine.

[12] Department of Surgery: Ajou University School of Medicine.

[13] Department of Surgery: Samsung Medical Center, Sungkyunkwan University School of Medicine.

[14] Konkuk University School of Medicine, Department of Nephrology .

[15] Department of Nephrology, Yeungnam University Hospital.

[16] Division of Nephrology, Department of Internal Medicine, Eunpyeong St. Mary's hospital.

[17] Department of Internal Medicine, Pusan National University Hospital.

[18] Department of Surgery: Ewha Womans University Seoul Hospital.

[19] Kosin University College of Medicine, Department of Internal Medicine, Division of Nephrology .

[20] Division of Nephrology, Department of Internal Medicine, Seoul St. Mary's hospital.

[21] Department of Internal Medicine, Incheon St. Mary’s Hospital, College of Medicine, The Catholic University of Korea College of Medicine.

[22] Department of Nephrology, Chungnam National University Hospital.

[23] Division of Nephrology, Department of Internal Medicine, Maryknoll Medical Center.

[24] Division of Nephrology, Department of Internal Medicine, Pusan National University School of Medicine.

[25] Department of Internal Medicine, Kangdong Sacred Heart Hospital, Hallym University College of Medicine.

[26] Division of Nephrology, Department of Internal Medicine, College of Medicine, Kyung Hee University.

[27] Division of Nephrology, Department of Internal Medicine, CHA Bundang Medical Center, CHA University, Seongnam, Korea.

[28] Department of Surgery: SMG-SNU Boramae Medical Center.

[29] Department of Surgery: Myongji Hospital.

[30] Department of Internal Medicine, Soonchunhyang University Seoul Hospital.

[31] Division of Nephrology, Inje University Ilsan-Paik Hospital.

[32] Division of Nephrology, Department of Internal Medicine, College of Medicine, Kyung Hee University Hospital, Kyung Hee University.

[33] Department of Surgery: Ulsan University Hospital.

[34] Department of Surgery: Gangnam Severance Hospital, Yonsei University College of Medicine.

[35] Department of Internal Medicine, Seoul National University Bundang Hospital.

[36] Department of Surgery: Chonnam National University Medical School.

[37] Department of Surgery: Asan Medical Center.

[38] Department of Internal Medicine, Keimyung University School of Medicine, Daegu, Korea.

[39] Department of Transplantation Surgery, Severance Hospital, Yonsei University College of Medicine.

[40] Department of internal medicine of nephrology, Inha university hospital, The Inha University of Korea, College of Medicine.

[41] Department of Surgery: Seoul National University Hospital.

[42] Department of Surgery: Dong-A University College of Medicine, Busan, Korea.

[43] Division of Nephrology, Korea University, College of Medicine, Guro Hospital.
